# The Ferric uptake regulator (Fur) and iron availability control the production and maturation of the antibacterial peptide microcin E492

**DOI:** 10.1371/journal.pone.0200835

**Published:** 2018-08-02

**Authors:** Andrés E. Marcoleta, Sergio Gutiérrez-Cortez, Felipe Hurtado, Yerko Argandoña, Gino Corsini, Octavio Monasterio, Rosalba Lagos

**Affiliations:** Departamento de Biología, Facultad de Ciencias, Universidad de Chile, Casilla, Santiago, Chile; Centre National de la Recherche Scientifique, Aix-Marseille Université, FRANCE

## Abstract

Microcin E492 is a pore-forming bacteriocin with toxic activity against *Enterobacteriaceae*, which undergoes amyloid aggregation as a mechanism to regulate its toxicity. To be active, it requires the posttranslational attachment to the C-terminus of a glycosylated enterochelin derivative (salmochelin), a process carried out by the proteins MceC, MceI and MceJ encoded in the MccE492 gene cluster. Both microcin E492 and salmochelin have a proposed role in the virulence of the bacterial pathogen *Klebsiella pneumoniae*. Besides, enterochelin is produced as a response to low iron availability and its synthesis is controlled by the global iron regulator Fur. Since the production of active microcin E492 depends on enterochelin biosynthesis, both processes could be coordinately regulated. In this work, we investigated the role of Fur in the expression of the microcin E492 maturation genes *mceCJI*. *mceC* was not *regulated* by Fur as it occurs with its homolog *iroB* in *Salmonella enterica*. We demonstrated that *mceJI* along with the previously uncharacterized gene *mceX* are transcribed as a single mRNA, and that Fur binds *in vivo* to a Fur box located upstream of the *mceX*-*mceJI* unit. Also, we established that the expression of these genes decreased in a condition of high iron availability, while this effect is abrogated in a Δ*fur* background. Furthermore, our results indicated that MceX acts as a negative regulator of microcin E492 structural gene expression, coupling its synthesis to the iron-dependent regulatory circuit. Consequently, *fur* or *mceX* overexpression led to a significant decrease in the antibacterial activity of cells producing microcin E492. Altogether these results show that both the expression of microcin E492 maturation genes *mceJI*, and MceX the negative regulator of microcin E492 synthesis, are coordinated with the enterochelin production by Fur, depending on the iron levels in the medium.

## Introduction

Microcin E492 (MccE492) is an ~8-kDa pore-forming bacteriocin that is produced and excreted by *Klebsiella pneumoniae* RYC492 [1–2]. Among its properties, MccE492 has antibacterial activity against *Enterobacteriaceae*, induces apoptosis in some human tumor cell lines, and forms amyloid fibers as a mechanism to regulate its activity [3–5]. Once synthesized, it undergoes posttranslational modification (maturation) by the attachment to the C-terminus of glycosylated derivatives of enterochelin (salmochelin), a siderophore widely used by *Enterobacteriaceae*. The addition of this moiety is essential for antibacterial activity, since it mediates the toxin recognition and internalization to the periplasm by the ferric catecholate receptors FepA, Fiu and Cir, located in the outer membrane of target cells [6–8]. Although not demonstrated experimentally, it was proposed that production of MccE492 and salmochelin would help to outcompete other bacteria during the colonization of iron-deprived environments, such as mammalian tissues. Moreover, it was found that genes involved in the production of these molecules are highly prevalent among hypervirulent *K*. *pneumoniae* strains [9–11], and thus likely play a role in the pathogenesis of this species, recently declared as an urgent pathogen trait due its hypervirulence and multi-drug resistance (reviewed in [12–13]).

The genetic determinants for the production of active MccE492 are grouped in a ~13-kbp gene cluster that was cloned and studied in *E*. *coli* [14]. It comprises (among others) the *mceA* and *mceB* genes coding for MccE492 peptide and its immunity protein, respectively; *mceH* and *mceG* coding for a dedicated ABC transporter and its accessory protein, and *mceC*, *mceJ* and *mceI*, which products are involved in MccE492 maturation; mutants in any of the maturation genes produce inactive MccE492 [15–17]. MceC protein is a 370-amino acid glycosyltransferase that catalyzes the addition of glucose moieties to enterochelin, forming salmochelin [16]. MceJ and MceI proteins are assembled in a complex that catalyzes the covalent attachment of salmochelin to the C-terminal end (serine 84) of MccE492 [16,18].

Bacteria, as most of living forms, need ferrous iron (Fe^2+^) to carry out many metabolic processes. Consequently, one of the first host’s defense barriers against pathogen infections consists of specialized proteins that sequester this micronutrient, reducing its bioavailability. In response, bacteria have developed siderophore-mediated iron uptake systems to circumvent this host defense strategy [19,20]. In *E*. *coli*, Ferric Uptake Regulator (Fur) protein is a masterpiece regulating cellular Fe^2+^ levels [19,21–22]. Fur acts mainly as a transcriptional repressor and senses intracellular iron availability: at high levels, Fe^2+^ acts as a co-repressor forming a Fur-Fe^2+^ complex that binds to a 19-bp sequence denominated Fur box, usually located in the promoter region of iron-regulated genes [23,24–25]. In contrast, at low Fe^2+^ levels, the Fur-Fe^2+^ complex is disassembled clearing the promoter region and allowing gene expression. Fur-mediated regulation controls the expression of more than 90 genes [26], including some virulence factors such as hemolysin and Shiga-like toxin, and the determinants coding for enterochelin synthesis [24,27–28]. As the precursors of MccE492 posttranslational modification are affected by the siderophore availability, and consequently by the iron uptake process, it is plausible that iron metabolism plays a role regulating MccE492 maturation. In this regard, previous work showed that active MccE492 production necessarily requires enterochelin as substrate [17]. It is also known that *iro*B gene, coding for the MceC homolog in *Salmonella*, has a Fur box in its promoter region that allows Fur-mediated and iron-dependent regulation of its expression [20,29–30]. Although these facts suggest that the MccE492 maturation process is connected with the iron-metabolism, the direct effect of Fur and iron availability on the expression of the MccE492 genetic determinants has not been studied. In this work we established that unlike its homolog *iroB*, *mceC* is not regulated by Fur. We characterized the promoter region of the *mceJI* maturation genes and found that they are transcribed as a polycistronic mRNA along with a small open reading frame named *mceX*, located upstream of *mceJ*. The promoter region driving the expression of this transcriptional unit harbors a Fur box overlapping the transcriptional start site, which binds Fur regulator *in vivo*. Using *lacZ* reporter fusions we provided evidence indicating that expression of *mceX* and *mceJI* is down-regulated by increasing iron concentrations in a Fur-dependent mechanism, and we established that MceX acts as a negative regulator of the *mceBA* unit, coupling the MccE492 protein expression to the iron regulation circuit. Consequently, *fur* or *mceX* overexpression led to a significant decrease in the antibacterial activity of cells producing MccE492, supporting the close relationship between both regulators and the biosynthesis and maturation of this bacteriocin.

## Materials and methods

### Bacterial strains and plasmids

The bacterial strains and plasmids used in this work are described in Tables 1 and 2, respectively.

**Table 1:** Bacterial strains used in this work.

*E*. *coli* **strain**	**Genotype**	**Source**
BL21 (DE3)	F^−^*ompT gal dcm lon hsdS*_*B*_(*r*_*B*_^-^ *m*_*B*_^-^) λ(DE3 [*lacI lacUV5-T7 gene 1 ind1 sam7 nin5*])	Novagen
MC4100	F^−^ (*araD139*) Δ(*argF-lac*)169 λ− e14− *flhD5301* Δ(*fruK-yeiR*)725(*fruA25*) *relA1 rpsL150*(Str^r^) *rbsR22* Δ(*fimB-fimE*)632(::IS*1*) *deoC1*	[63]
H1941	MC4100-derivative. *fur*-	[64]
DH5α	*sup*E44 Δ*lac*U169 (Φ80*lac*ZΔM15) *hsd*R17 *rec*A1 *end*A1 *gyr*A96 *thi*-1 *rel*A1.	Promega
H1717	F^-^ araD139 Δ*lac*U169 *rps*L *rel*A *thi*A, Mot^-^ *fhu*A::λplacMu	[36]

**Table 2:** Plasmids used in this work.

**Plasmid**	**Description**	**Source**
p15A-*fur*	pACYC184-derived. Harbors a *fur* expression cassette that was PCR-amplified from pFur and cloned in the *Eco*RV site. (Cm^r^)	This work
pT5-*mceX*	P15A origin, *lacI*^q^. Harbors the *mceX* gene under the control of a T5 promoter regulated by two *lacO* operator sequences (IPTG-inducible). (Cm^r^)	This work
pACYC184	General purpose plasmid with p15A replication origin. (Cm^r^, Tet^r^).	[65]
pCA24N	Plasmid with ColE1 replication origin. Harbors a T5-lac promoter and a His-6x to allow the expression of recombinant proteins (IPTG-inducible). (Cm^r^)	[66]
pCR4-TOPO	pUC-derived plasmid for topoisomerase-mediated TA cloning (Invitrogen). (Amp^r^, Kan^r^).	Invitrogen
pFur	pCA24N-derived. Harbors *fur* gene fused to a N-terminal His-tag, to be expressed under the control of T5-lac promoter (IPTG-inducible). (Cm^r^)	ASKA collection
pFurC	pACYC184-derived. Harbors a 346 bp fragment from the promoter and coding region of *mceC*.	This work
pFurD	pACYC184-derived. Harbors a 268 bp fragment from the promoter and coding region of *mceD*.	This work
pFurE	pACYC184-derived. Harbors a 240 bp fragment from the promoter and coding region of *mceE*.	This work
pFurX	pACYC184-derived. Harbors a 237 bp fragment from the promoter and coding region of *mceX*.	This work
pHC79	ColE1-derived cosmid. General purpose. (Amp^r^, Tet^r^).	[67]
pHF	pACYC184-derived. Harbors a 3.7-kbp fragment from the middle of *mceH* gene to the start of *mceF* gene.	This work
pJAM229	pHC79-derived, harbors MccE492 production cluster with a truncated version of *mceF* gene.	[14]
pJAM434	pHC79-derived, harbors MccE492 production cluster with a truncated version of *mceF* gene and an inversion of an internal *Xho*I segment (compared to the orientation found in the *K*. *pneumoniae* RYC492 chromosome.	[14]
pJI	pJAM434-derivative with a 6.8-kbp BstVI deletion. Harbors *mceABCDE* genes. (Amp^r^).	[14]
pJIE291	pJAM434-derivative, formed by the re-ligation of 3 *Eco*RI fragments. Harbors *mceCDE* genes. (Amp^r^).	[14]
pMAH34	pACYC184-derivative harboring a PCR-amplified fragment comprising *mceA* and *mceB* genes with their natural promoter. (Cm^r^).	This work
pMccE492	pJAM229 derivative. Harbors MccE492 production cluster with complete *mceF* gene (Amp^r^).	[68]
p*mceBA*’-’*lacZ*	pACYC184-derived. Harbors a translational fusion between the last amino acid of MceA and LacZ. (Cm^r^).	This work
p*mceC*’-’*lacZ*	pACYC184-derived. Harbors a translational fusion between the last amino acid of MceC (Gln 370) and LacZ. (Cm^r^).	This work
p*mceJ*17’-’*lacZ*	p*mceJI*’-’*lacZ*-derived plasmid. Harbors a translational fusion between the 17^th^ codon of *mceJ* and *lacZ*. (Cm^r^).	This work
p*mceJI*’-’*lacZ*	pACYC184-derived plasmid. Harbors a translational fusion between leucin 183 of MceI and LacZ. (Cm^r^).	This work
p*mceJI*ΔTSS’-’*lacZ*	pACYC184-derived plasmid. Harbors a translational fusion between leucin 183 from MceI and LacZ. It lacks the region between -177 and +123 respect of the *mceJI* unit transcription start site. (Cm^r^).	This work
p*mceX*’-‘*lacZ*	p*mceJI*’-‘*lacZ*-derived plasmid. Harbors a translational fusion between the last codon of *mceX* and *lacZ*. (Cm^r^).	This work
pPelican	pUC8-derived reporter plasmid. Harbors a *lacZ* gene expression cassette flanked by *gypsy* transposon insulator sequences.	[69]
pXH	pACYC184-derived plasmid. Harbors a 3.5-kbp fragment from *mceX* start codon to the middle of *mceH* gene.	This work

### Growth conditions

Bacterial growth was performed diluting 1,000–2,000X an overnight culture in M9 medium supplemented with citrate and glucose (2 g/L each), or in Luria Broth (LB, Difco), and incubating at 37 °C with shaking (180 rpm). When required, antibiotics were used at the following concentrations: Ampicillin (Amp) 100 μg/mL, kanamycin (Kan) 50 μg/mL, tetracycline (Tc) 50 μg/mL, chloramphenicol (Cm) 50 μg/mL.

### Recombinant DNA techniques

DNA extraction, ligation, digestion with restriction enzymes and other routine procedures not further detailed, were performed according to standard protocols [31], and manufacturer’s guidelines. *E*. *coli* strain DH5α was used to maintain and propagate DNA constructs.

### MccE492 activity assay on plates

Antibacterial activity determination in plates was performed mixing an aliquot of 0.3 mL of ~1 x 107 cells/mL of the *E*. *coli* indicator strain with 3 mL of M9 soft agar (0.7% w/v agar), and overlaying onto plates containing M9 medium supplemented with glucose and citrate. MccE492 antibacterial activity was detected by the formation of growth inhibition halos. Halo’s area was measured (in square pixels) using ImageJ software [32], and then was normalized to a reference condition. A lawn of the sensitive *E*. *coli* BL21(DE3) was routinely used as the indicator strain.

### *In silico* identification of regulatory elements upstream of maturation genes

Promoter search was performed using BPROM application, publicly available at http://linux1.softberry.com [33].

### RNA extraction

RNA extraction was performed as described previously [34], with minor modifications. Briefly, a volume corresponding to OD_600_ = 4.0 of a bacterial culture was mixed with 1/5 volume of cold stop solution (5% phenol in ethanol), vigorously shaken, frozen in liquid nitrogen and stored at -80 °C until processing. Samples were thawed in ice and centrifuged for 10 min at 17,000 x g (4 °C). After discarding the supernatant, the bacterial pellet was suspended in 1 mL of TRIzol (Ambion), and the mixture was transferred to a PLHG phase separation tube (Phase Lock Heavy Gel, Eppendorf). Then, 400 μl of CH_3_Cl were added to the tube mixing vigorously during 10 s, incubated at room temperature during 5 min and centrifuged 1 min at 17,000 x g for phase separation. The aqueous phase was placed in a fresh tube, 1 volume of isopropanol was added and the mixture was incubated 30 min at room temperature. After centrifuging (30 min, 17,000 x g), the pellet was washed with 350 μl of 75% ethanol, and then dissolved in nanopure water. RNA concentration and purity (A_260_/A_280_ ratio) were determined using a Nanodrop spectrophotometer (Thermo Scientific).

### RT-PCR

Prior to cDNA synthesis, total RNA samples were treated with DNAse I (Fermentas) according to the manufacturer’s protocol. After incubation, DNAse I was removed extracting with 1 volume of P:C:I (phenol/chloroform/isoamylic alcohol, 25:24:1 v/v), and then precipitating the RNA by adding 1/10 volumes of 3M sodium acetate (pH 5.7) and 4 volumes of 100% ethanol, and incubating 1 h on ice. After centrifugation, the supernatant was discarded and the pellet was washed once with 75% ethanol, and dissolved in nanopure water.

Reverse transcription reactions were performed using SuperScript III reverse transcriptase (Life Technologies), following the manufacturer’s instructions. Each reaction was prepared with 2 μg of DNA-free total RNA and 2 pmol of the corresponding primer. Negative control reactions were performed replacing reverse transcriptase by nanopure water. After the first strand synthesis, samples were incubated with 1 U of RNase H (Invitrogen) during 20 min (37 °C), and stored at -20 °C until used. PCR amplification was done with 2 μL of each prepared cDNA, 1X *Taq* polymerase buffer, 0.25 mM dNTPs, 0.5 pmol of each specific primer and 0.025 U of *Taq* polymerase. For *mceX*/*mceJI* amplification the primers used were JVO-5475/JVO5476 (*mceX*), JVO-5475/JVO-5477 (*mceX*-*mceJ*), and JVO-5478/JVO-5479 (*mceJ*-*mceI*). Thermal profile for amplification cycles was 95 °C/10 min, 35 x (95 °C/40 s, 57 °C/40 s, 72 °C/45 s), 72 °C/10 min. The amplification products were detected by 2% agarose gel electrophoresis and ethidium bromide staining.

### 5’ RACE assay

The determination of the transcription start site for the *mceX*/*mceJI* transcriptional unit by 5’ RACE assay was performed following the general strategy described by Urban and Vogel [35]. Briefly, total RNA was extracted from late-exponential cultures of *E*. *coli* VCS257 carrying pJAM229 plasmid, grown in glucose/citrate-supplemented M9 minimal medium (OD_600_ = 0.8). Contaminating DNA was removed by DNAse I treatment (as described in RT-PCR method). Then, 12 μg of DNA-free RNA were mixed with 10 μL of 10X TAP buffer and RNAse-free water, completing a total volume of 98 μL. This mixture was divided in two tubes, and then 10 units of TAP (Epicentre Technologies) were added to one, and an equivalent volume of water was added to the control. Samples were incubated at 37 °C during 30 min. After incubation, 300 pmol of the A4 RNA adaptor (see Table 3) were added, and the RNA mixture was further purified by P:C:I extraction and ethanol precipitation. Subsequently, the RNA pellet was suspended in RNAse-free water, and RNA-adaptor ligation was performed using 40 U of T4 RNA ligase (New England Biolabs), following the manufacturer’s instructions. After a second round of P:C:I extraction and ethanol precipitation, the resulting RNA was used for cDNA synthesis as described in RT-PCR method section, using 100 pmol of random hexamers as primers. PCR amplification from the cDNA prepared above was performed using primers JVO-5476 (*mceX*) and JVO-5477 (*mceJ*), in combination with a specific primer directed to A4 RNA-adaptor (JVO-0366). PCR amplicons were further visualized in a 2% agarose gel electrophoresis. For sequence analysis, selected DNA bands were excised from gel, purified using the Quickspin kit (Qiagen), and cloned into pCR4®-TOPO®, following manufacturer’s guidelines. Sequencing was performed using the universal M13F and M13R primers.

**Table 3:** List and sequence of primers used in this work.

Primer name	Sequence (5’ → 3’)	Features/purpose
A4	GACGAGCACGAGGACACUGACAUGGAGGAGGGAGUAGAAA	RNA adaptor
C-Fus-F	ACAGGAGAAATCTAGAGGCCGGCCAATGTCATAAGTTTTATCCGTTA	*Fse*I/p*mceC*’-’*lacZ* construction
C-Fus-R	TTCAGCAGTGGCGCGGCCGCTACCTTGCCAGATGGTTTTCAGTTT	*Not*I/p*mceC*’-’*lacZ* construction
FurC	CCTGTGTCAGGCATAATTA	*mceC* Fur box
FurD	GGGCTACGCGGCTCTGCT	*mceD* Fur box
FurE2	CCAATGTGCTCACTCAGCATA	*mceE* Fur box
FurX	GAC TTC GTG TGT CTA ATA TGT CA	*mceX* Fur box
JI229-Fus-F	AGCTTTGTTGGGCCGGCCTTCAGTCTCCCATCAGTTAA	*Fse*I/p*mceJI*’-‘*lacZ* construction
JI229-Fus-R	GGGTACATTTCGGAGCGGCCGCGCCAAAGTTCTTTCTGTGTCTCCG	*Not*I/p*mceJI*’-‘*lacZ* construction
JI434-Fus-F	AGCTTTGTTGGGCCGGCCCTCGAGTATTCTTTGTCAAT	*Fse*I/p*mceJI*ΔTSS’-‘*lacZ* construction
JVO-0366	GGACACTGACATGGAGGAG	Directed to A4 RNA-adaptor
JVO-5475	GGTGTTTGCTACAGACCTG	*mceX*/RT-PCR
JVO-5476	CAGAACATTGACAAAGAATAC	*mceX*/5’RACE
JVO-5477	GATAGTTACCATCAAATCCAC	*mceJ*/5’RACE
JVO-5478	GCGATAACCCCTATTACCTG	*mceJ*/RT-PCR
JVO-5479	CCATCGGCATTTTATGTCG	*mceI*/RT-PCR
JVO-5481	P~CCCGTCGTTTTACAACGTC	p*mceX*’-‘*lacZ* construction
LACLG_ASKA_F	CGTGTTGAGTGCCATCGATGGAAAACCTTTCGCGGTATGG	*Cla*I/p15A-*fur* construction
LACLQ2_ASKA_R	CTACTGACGGGGTGGTGCGTAAC	p15A-*fur* construction
LacZ-Pelic-F	GCTGACACAAGCGGCCGCGAACCCGTCGTTTTACAACGTC	*Not*I/*lacZ*
LacZ-Pelic-R	CGGTACTTCAGGCGCGCCACCTTATTTTTGACACCAGACCA	*Asc*I/*lacZ*
Mcc-Fus-F	ACAGGAGAAATCTAGAGGCCGGCCGGCCAATAAAGAGAATACGC	*Fse*I/p*mceBA*’-‘*lacZ* construction
Mcc-Fus-R	TTCAGCAGTGGCGCGGCCGCTACCACTACCACTACCGGAACTGG	*Not*I/p*mceBA*’-‘*lacZ* construction
mceFReFW1	GCCCGGAAATTATGTCTCGAGGACT	pHF construction
mceHReFW1	AATGGTCAATGCCGGAGACAGT	pHF construction
mceHReRV1	TAGGTCAGCATTTCCTGCGTTGAG	pXH construction
mceX-Fus-R	GAACATTGACAAAGAATACTCG	p*mceX*’-‘*lacZ* construction
mceXReFW1	ATGGTGTTTGCTACAGACCTGGTG	pXH construction
PEC	CTTCATGTCCGTTCACACGA	*mceC* Fur box
PED	TGTGGACAACTGTGCACATAT	*mceD* Fur box
PEE	GGAATAGAGGAGAGTGAGGA	*mceE and mceX* Fur boxes
PEX	GGGATCCTATACGGAATAAGATC	*mceX* Fur box

Restriction sites are underlined.

### Fur titration assays (FurTA)

Detection of Fur *in vivo* binding to putative Fur boxes was performed using the FurTA assay described previously [36]. Briefly, *E*. *coli* H1717 strain harboring a chromosomal copy of the Fur-regulated *fhuF* promoter fused to *lacZ* gene, were transformed with multi-copy plasmids carrying DNA segments to be tested. The resulting strains were then plated into MacConkey agar plates supplemented with 60 μM FeSO_4_, and incubated overnight at 37°C. Orange to red coloration of the colonies indicated that the tested DNA segment has a functional Fur box that can sequester Fur, preventing its binding to the *fhuF* promoter and allowing the expression of the *lacZ* gene, resulting in the coloring of the colonies.

Constructs pFurC, pFurD, pFurE, pFurX, pXH and pHF comprising distinct regions of MccE492 gene cluster to be tested, were generated amplifying each region by PCR using *Pfu* DNA polymerase and cloning the amplicons into the *Eco*RV site of pACYC184. To generate pFurC, a 346 bp fragment was amplified using FurC and PEC primers. pFurD was constructed cloning a 268 bp fragment amplified using FurD and PED primers. pFurE was constructed cloning a 240 bp fragment amplified using PEE and FurE2 primers. pFurX was constructed cloning a 237 bp fragment amplified using FurX and PEX primers. pXH was constructed cloning into pACYC184 a 3.5-kbp DNA fragment amplified by PCR using primers mceXReFW1 and mceHReRV1. Finally, to construct pHG, a 3.7-kbp fragment was PCR-amplified using primers mceHReFW1 and mceFReFW1.

### Construction of *lacZ* reporter fusions

LacZ fusions were constructed amplifying by PCR the desired region of the MccE492 genetic cluster using primers harboring *Fse*I or *Not*I restriction sites, and then ligating it to a pACYC184 backbone, and to a *lacZ* gene amplified from pPelican plasmid using primers LacZ-Pelic-F and LacZ-Pelic-R. The genes *mceBA*, *mceC*, and *mceJI* (including a ~500-bp region upstream of the start codon) were amplified using pJAM229 as template DNA, and primers Mcc-Fus-F and Mcc-Fus-R (*mceBA*), C-Fus-F and C-Fus-R (*mceC*), or JI229-Fus-F and JI229-Fus-R (*mceJI*). This way, we generated plasmids p*mceBA*’-‘*lacZ*, p*mceC*’-‘*lacZ* and p*mceJI*’-‘*lacZ*. Alternatively, primers JI434-Fus-F/JI229-Fus-R were used to generate p*mceJI*ΔTSS’-‘*lacZ* plasmid. p*mceX*’-‘*lacZ*, was constructed following an inverted PCR approach, starting from p*mceJI*’-‘*lacZ*. Briefly, divergent PCR primers mceX-Fus-R and JVO5481 (phosphorylated at its 5’ end) and the high fidelity Phusion Polimerase (New England Biolabs) were used to PCR amplify the region of the plasmid to be maintained. Then, the template plasmid was removed by *Dpn*I digestion, and the PCR product was self-ligated and transformed into TOP10 *E*. *coli* cells. p*mceJ*17’-‘*lac*Z was obtained through the mutagenesis service offered by Genscript Inc., starting from p*mceJI*’-‘lacZ.

### p15A-*fur* and pT5-*mceX* plasmid construction

To clone the IPTG-inducible Fur expression cassette in a plasmid compatible with pMccE492 or pJAM434 (allowing the production of MccE492 with different degrees of antibacterial activity), a ~2,5-kbp PCR fragment comprising this cassette and *lacI*^*q*^ (lac repressor) were amplified from pFur using LACLG_ASKA_F and LACLQ2_ASKA_R primers, and cloned in the EcoRV site of pACYC184, disrupting the tetracycline-resistance gene. In order to generate pT5-*mceX*, an expression cassette was designed and synthesized (Genscript Inc.). This cassette comprises the consensus sequences specifying a T5 promoter, a ribosomal binding site, the *mceX* coding region, and a T5 terminator. Once synthesized, the cassette was subcloned into the p33AM plasmid backbone, encoding the LacI^q^ repressor.

### β-galactosidase activity determinations

The method used was similar to that described originally by Miller [37]. For each sample, about 2 mL of bacterial culture were collected in a pre-chilled tube, the optical density at 600 nm was measured, and the medium was removed by centrifugation. Bacterial pellets were stored at -80 °C until processed. For activity determination, cell pellets were ice-thawed and resuspended in 1 mL of Z buffer (60 mM Na_2_HPO_4_, 40 mM NaH_2_PO_4_·2H_2_O, 10 mM KCl, 1 mM MgSO_4_·7H_2_O, 50 mM β-mercaptoethanol), incubated at 30 °C during 5 min, and then 200 μL of ONPG (4 mg/mL in Z buffer without β-mercaptoetanol) were added starting the count of reaction time. When a strong yellow color was observed (OD_420_ = 0.1–0.5), the reaction was stopped adding 500 μL of 1 M Na_2_CO_3_, and the elapsed time from the start point was recorded. Samples were centrifuged at 17,000 x g during 2 min and the absorbance at 420 nm of supernatant was measured. β-galactosidase activity expressed in Miller units was calculated using the following formula:

$$\mathrm{Miller}\;\mathrm{units}=\left({\mathrm{OD}}_{420}\;*\;1000\right)/\left({\mathrm{OD}}_{600}\;*\;V\;*\;t\right),\;\mathrm{where}\;V=\mathrm{volume}\;\mathrm{of}\;\mathrm{culture}\;\mathrm{processed}\;\mathrm{for}\;\mathrm{each}\;\mathrm{sample},\;\mathrm{and}\;t=\mathrm{time}\;\mathrm{elapsed}\;\mathrm{from}\;\mathrm{start}\;\mathrm{to}\;\mathrm{end}\;\mathrm{of}\;\mathrm{the}\;\mathrm{reaction}.$$

### Protein total extracts preparation, SDS-PAGE and immunoblot

For total protein extraction preparations, an amount of cells equivalent to OD_600_ = 1 was collected in a pre-chilled tube by centrifugation at 17,000 x g during 2 min (4 °C). Pellets were suspended in 100 μL of 1X loading buffer (63 mM Tris-HCl, 10% glycerol, 2% SDS, 0.0025% bromophenol blue, 5% β-mercaptoethanol, pH 6.8). Samples were heated to 95 °C for 5 min before electrophoresis analysis. 10 μL of the extracts were analyzed by SDS-PAGE, in a 15% polyacrilamide gel. Protein bands visualization was performed with conventional Coomassie blue G25 staining procedures. Alternatively, proteins were transferred from the gel to a nitrocellulose membrane using a blotting transfer unit filled with transfer buffer (25 mM Tris-HCl, 190 mM glycine, 20% methanol), applying a constant current of 400 mA during 90 min. Immunoblot detection was performed as reported previously [17], using an anti-His antibody (Santa Cruz Biotechnology) or an anti-GroEL antibody (Sigma) as required, and a secondary antibody coupled to peroxidase (Pierce).

### Statistical analyses

Statistical analysis to evaluate significance of the differences observed from the data sets showed in Figs 3 and 4 were performed using Two-way ANOVA with a Sidak’s multiple comparison test to evaluate relevant pairs of conditions. For Figs 5 and 6, one-way ANOVA with a Bonferroni’s multiple comparison test was used. All the calculations were done using the Graphpad Prism 6 software.

## Results

### Fur-binding motifs are present in the promoter region of the microcin E492 maturation genes

**Fig 1:**
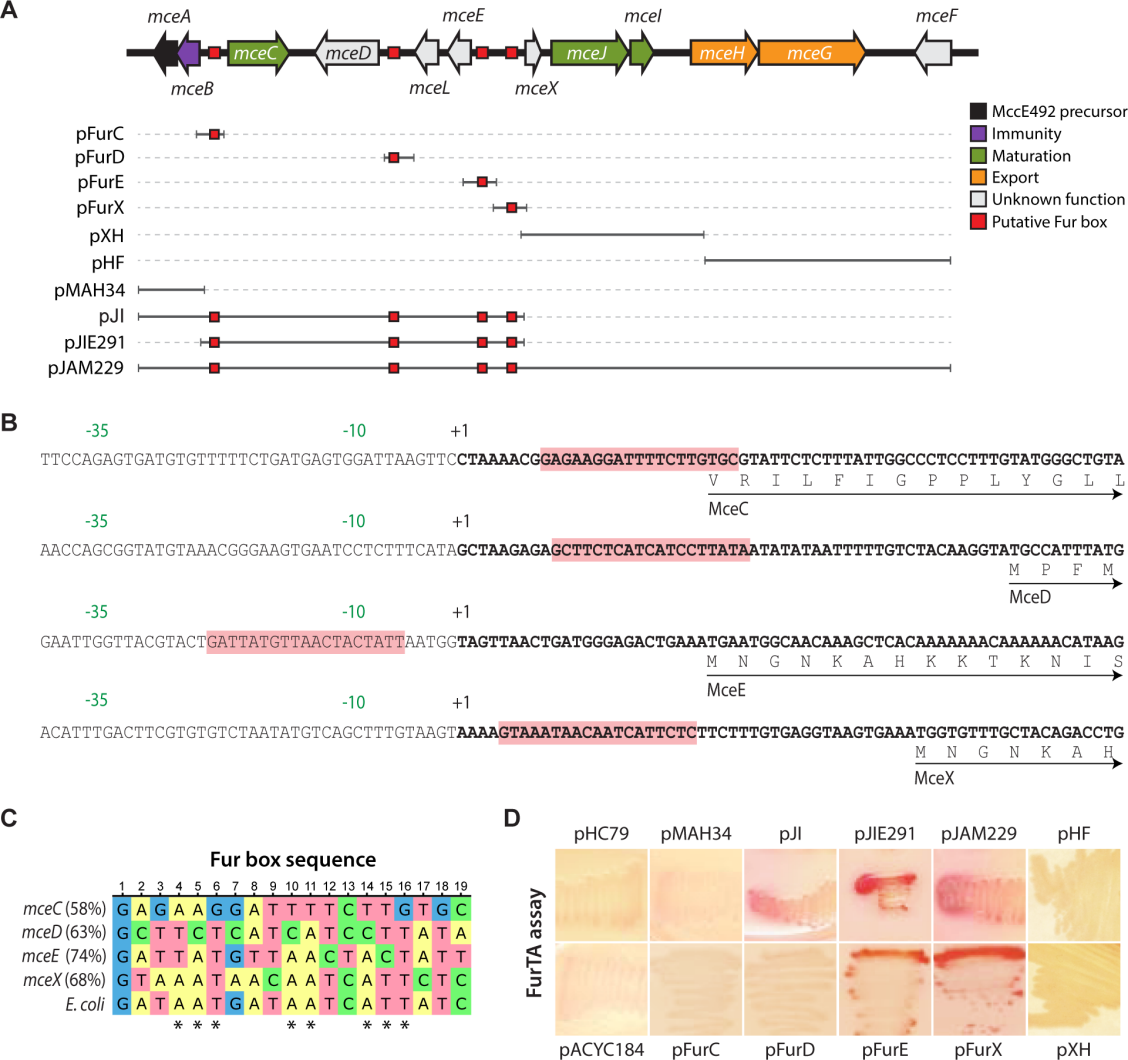
Functional Fur boxes are located upstream of the microcin E492 maturation genes *mceJI*. (A) Sequence analysis revealed the presence of putative Fur boxes in the MccE492 gene cluster (red boxes). Solid gray segments represent the regions of the cluster that were cloned in the respective plasmid listed in the left side, and that were used in the FurTA assays showed in (D). (B) Nucleotide sequence of the 5’ upstream regions from genes *mceC*, *mceD*, *mceE* and *mceX*. Experimentally determined transcription start site for each gene is indicated as “+1”, while -35 and -10 promoter elements are shown in green. The identified putative Fur boxes are shaded in red. (C) Nucleotide sequence aligment of the Fur boxes identified in the promoter regions of *mce*C, *mceD*, *mceE* and *mceX* genes, as well as the consensus sequence previously defined for *E*. *coli*. A different color was assigned to each nucleotide base. The percentage identity of each Fur box with the consensus sequence is showed in parenthesis. The most conserved nucleotide positions of the *E*. *coli* consensus are marked with asterisks. (D) FurTA assay results for *E*. *coli* H1717 reporter strain transformed with one of the plasmids listed in (A), or with the control plasmids pHC79 and pACYC184. Red coloration after growing in iron-supplemented MacConkey agar plates indicates Fur binding *in vivo*.

**Fig 2:**
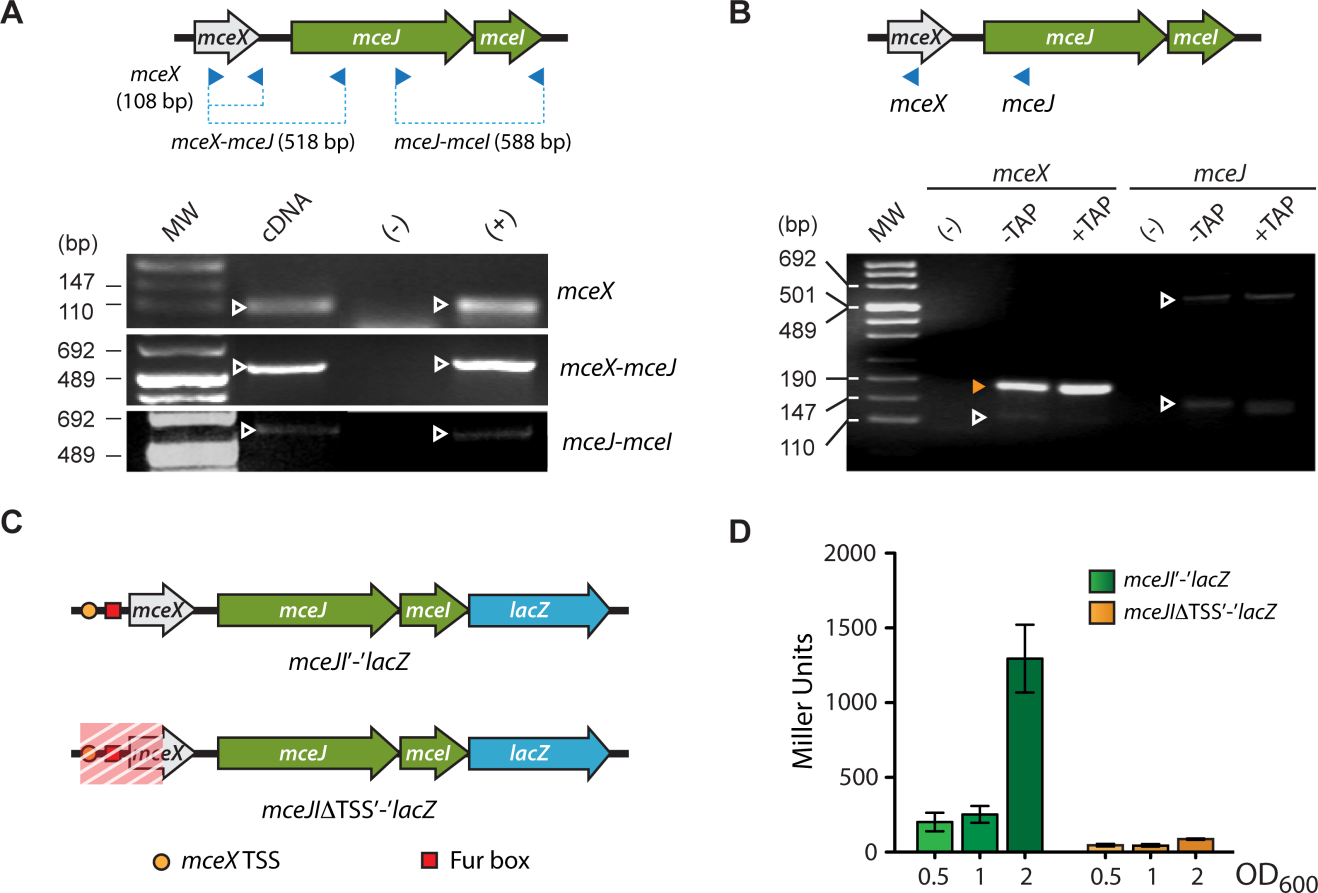
*mceX*, *mceJ* and *mceI* genes are transcribed as a single mRNA from a promoter located upstream of the *mceX* Fur box. (A) RT-PCR detection of a polycistronic mRNA harboring *mceX*, *mceJ* and *mceI*. The cDNA was prepared from total RNA of *E*. *coli* cells transformed with pJAM229. The amplicons originated from each primer pair are represented in the scheme as blue dashed lines, and are marked with white arrowheads in the corresponding gel section. Total RNA non-subjected to reverse transcription was used as negative control (-), while pJAM229 plasmid was used as positive control (+). (B) 5’ RACE assay to determine the transcription start site of the *mceX/mceJI* unit. Two primers were used (blue arrowheads), one hybridizing to *mceX* and the other hybridizing to *mceJ*. A band of 147–190 bp was enriched after TAP treatment when using *mceX* primer (orange arrowhead). Bands not enriched after TAP treatment were also observed using *mceX* or *mceJ* primers (white arrowheads). Total RNA non-subjected to reverse transcription was used as negative control (-). (C) Two reporter constructs were obtained fusing *lacZ* to the last codon of *mceI* gene. The *mceJI’-‘lacZ* construct harbors *mceJ*, *mceX*, and its upstream region (including the TSS). The *mceJIΔTSS’-‘lacZ* construct is identical to *mceJI’-‘lacZ*, except for lacking a region (shaded in red) comprising the *mceX* promoter and part of its coding region. (D) β-galactosidase activity was measured in *E*. *coli* cells transformed with each of the reporter fusions and grown until different optical density. Error bars correspond to standard deviation of 3 independent experiments.

To evaluate the possible Fur-dependent regulation of the MccE492 gene cluster, we performed an *in-silico* search for putative Fur binding motifs. Using the consensus sequence GATAATGATAATCATTATC previously described for *E*. *coli* [24,38], we identified putative Fur boxes upstream of the genes *mceC*, *mceD*, *mceE*, and *mceX* (Fig 1A and 1B, red boxes; Fig 1C). In a previous work we determined that *mceC*, *mceD*, and *mceE* genes are transcribed as monocistronic mRNAs, and their transcription start sites (TSS) were determined [15]. The putative Fur binding sequences for these genes were found overlapping the -10 promoter element in the case of *mceE*, and between -10 and the start codon for both, *mceC and mceD*. These three locations are compatible with Fur boxes present in upstream regions of already characterized Fur-regulated genes [38–39]. On the other hand, as the *mceX* TSS was not previously identified, it was determined in this work (Fig 2), indicating that the putative Fur box is located between -10 and the start codon of this gene (Fig 1B).

To gain information about the functionality of the putative Fur boxes identified inside the MccE492 genetic cluster, we evaluated their capability to bind Fur *in vivo* using the Fur titration assay (FurTA) described previously [36]. For this, a DNA segment containing one or more of the Fur boxes to be tested was cloned in a multi-copy plasmid and then transformed into the reporter strain H1717, carrying a chromosomally encoded fusion between the *fhu*F promoter (regulated by Fur) and the *lac*Z gene. When this reporter strain is transformed with a plasmid harboring a functional Fur box, Fur is titrated from the *fhu*F promoter. Consequently, *lacZ* is expressed and the colonies are visualized as red when grown over iron-supplemented MacConkey agar plates. If the DNA segment to be assayed lacks a functional Fur binding element, *lacZ* expression remains repressed and the transformed reporter strain shows no red coloration. The solid gray lines in Fig 1A represents the DNA segments from the MccE49 cluster that were cloned and transformed into the H1717 reporter strain, some of them harboring putative Fur boxes (red squares). After growing on iron-supplemented MacConkey agar plates, red coloration was evaluated for strains carrying each segment (Fig 1D). Only the segments carrying Fur boxes located in the *mce*E/*mce*X intergenic region showed to bind Fur *in vivo*. No red coloration was observed neither with the empty plasmid vectors (pACYC184 and pHC79) nor with plasmids carrying DNA segments from other regions of the MccE492 cluster, including those containing the putative Fur boxes located upstream of *mce*C and *mce*D genes. Thus, Fur regulator binds *in vivo* to the region upstream of *mce*X, suggesting a role of this regulator in controlling *mceX* and possibly *mceJI* expression. Accordingly, the alignment of the Fur boxes identified in the MccE492 genetic cluster indicated that *mceX* and *mceE* Fur boxes showed the highest identity with the consensus *E*. *coli* sequence (Fig 1C). Moreover, *mceX* Fur box showed a 100% conservation in the nucleotide positions proposed to be the most relevant for the binding of Fur regulator in Fur-repressed genes [25].

### Organization of the transcriptional units containing the microcin E492 maturation genes

In previous work using the pJAM434 plasmid, it was concluded that the maturation genes *mceJI* are co-transcribed along with the export genes *mceGH* [15,40]. However, lately it was noted that pJAM434 harbors a mutant version of the MccE492 gene cluster with an inverted 6.8-kbp *Xho*I fragment, as compared to the *wild type* orientation (reviewed in [3] and confirmed after *K*. *pneumoniae* RYC492 genome sequencing [41]). This inversion takes apart the *mceJI* genes from their natural 5’ upstream region, which contains at least two features: *mceX*, and the Fur box located upstream of *mceX* identified in this study. Hence, it was necessary to review the structure of the *mceJI* transcriptional unit and its promoter region. Likely, if the *mceJI* maturation genes were transcribed coupled to *mceX*, the identified functional Fur box would control the expression of all of them. Using *in silico* searches, no putative promoter elements were found in the region between *mceX* and *mceJ*, while putative -10/-35 elements were found in the region 47 to 86 bp upstream of the *mceX* start codon. To demonstrate the importance of this region in directing the transcription of *mceX* and its possible coupling to *mceJI*, we first searched for such polycistronic mRNA by RT-PCR analysis (Fig 2A). For this purpose, total RNA was extracted from an *E*. *coli* host transformed with pJAM229 plasmid containing the microcin production gene cluster (in the *wild type* orientation), which was used to synthesize cDNA by reverse transcription. Then, using distinct sets of oligonucleotide primers directed to *mceX*, *mceJ* and *mceI*, we were able to amplify cDNA segments bearing parts of both *mceX*/*mceJ* and *mceI*/*mceJ* coding regions. The results shown in Fig 2A indicate that *mceX* is transcribed, and the transcript extends downstream including the *mceJ* and *mceI* coding regions.

Next, we determined the transcription start site of the *mceX*-*mceJI* mRNA using a 5’RACE strategy [35]. In this method, primary transcripts can be enriched over processed or degraded mRNAs, using the Tobacco Acid Pyrophosphatase (TAP), which hydrolyzes the tri-phosphate moiety present only in primary transcripts, generating a 5’-phosphate end that allows the ligation of an RNA adapter of known sequence to this mRNA. After ligating the adaptor, RNA is retro-transcribed using random hexamers, and the resulting cDNAs are used as template for PCR amplification with an adaptor-directed primer and a second primer specific for the transcript whose TSS is being determined. PCR amplicons are resolved by agarose-gel electrophoresis, and the primary transcript can be identified as a band that is enriched after TAP treatment. This positive band is purified and sequenced, determining the first base next to the adaptor (corresponding to the TSS). The experiment was performed with RNA extracted from *E*. *coli* transformed with pJAM229, using two primers: one hybridizing 86 bp upstream of putative *mceX* start codon, and the other 253 bp downstream of *mceJ* start codon (Fig 2B, top). After PCR amplification and gel electrophoresis, a couple of bands were observed for both *mceX* and *mceJ* primers (Fig 2B, bottom). Among them, a 147-190-bp amplicon originated from *mceX* primer (orange arrowhead) was enriched after TAP treatment and considered positive for the 5’RACE assay. Sequence analysis of this band showed that the TSS corresponds to an “A”, 42 bp upstream of *mceX* start codon and 288 bp upstream *mceJ* start codon (Fig 1B). From this, the deduced -10 and -35 sites resemble the α^70^ consensus for these elements, and fall near the position predicted bioinformatically. With the *mceJ* primer, we observed a faint 500-700-bp band that fits the expected distance to the *mceX* TSS described above (563 bp). Unexpectedly, this band seems to be negative for TAP enrichment. This result can be explained as a consequence of the large size of the amplicon, which is undesired for TSS assessment by this technique [34]. A second amplicon, also very faint, was obtained with the *mceJ* primer and could be considered positive for TAP enrichment. This points out the possibility of a second TSS located in the *mceJ* coding region. However, neither putative ribosome binding sequences nor translation start sites could be identified downstream of this putative TSS.

To evaluate the functionality of the putative secondary TSS, and validate the *mceX* TSS identified as the primary start site, we constructed two reporter fusions: *mceJI’-‘lacZ* and *mceJIΔTSS’-‘lacZ* (Fig 2C). The former harbors a DNA segment containing 490 bp upstream of *mceJ* start codon (including the primary TSS, Fur box and *mceX*), the *mceJ* gene, and *lacZ* fused to the last codon of *mceI*. The latter has the same elements except for a deletion that starts 189 bp upstream of *mceJ* start codon, eliminating near half of the *mceX* coding region, the Fur box and the primary TSS. Both constructs were transformed in *E*. *coli* and LacZ activity was measured over bacterial growth, as a measure of *mceX*/*mceJI* expression (Fig 2D). Here, the expression levels of these genes decreased near to the detection limit in all the growth phases, when the region including the primary TSS was removed. This evidence supports the TSS of *mceX* as the primary transcription start site for the *mceX*-*mceJI* unit, and that the putative secondary TSS is poorly active under our experimental conditions.

Taken together, these results demonstrate that *mceX* and the maturation genes *mceJI* are co-transcribed sharing a common TSS located upstream of a functional Fur box.

### The expression of *mceX* and *mceJI*, but not *mceC* is regulated by iron availability through a Fur-dependent mechanism

**Fig 3:**
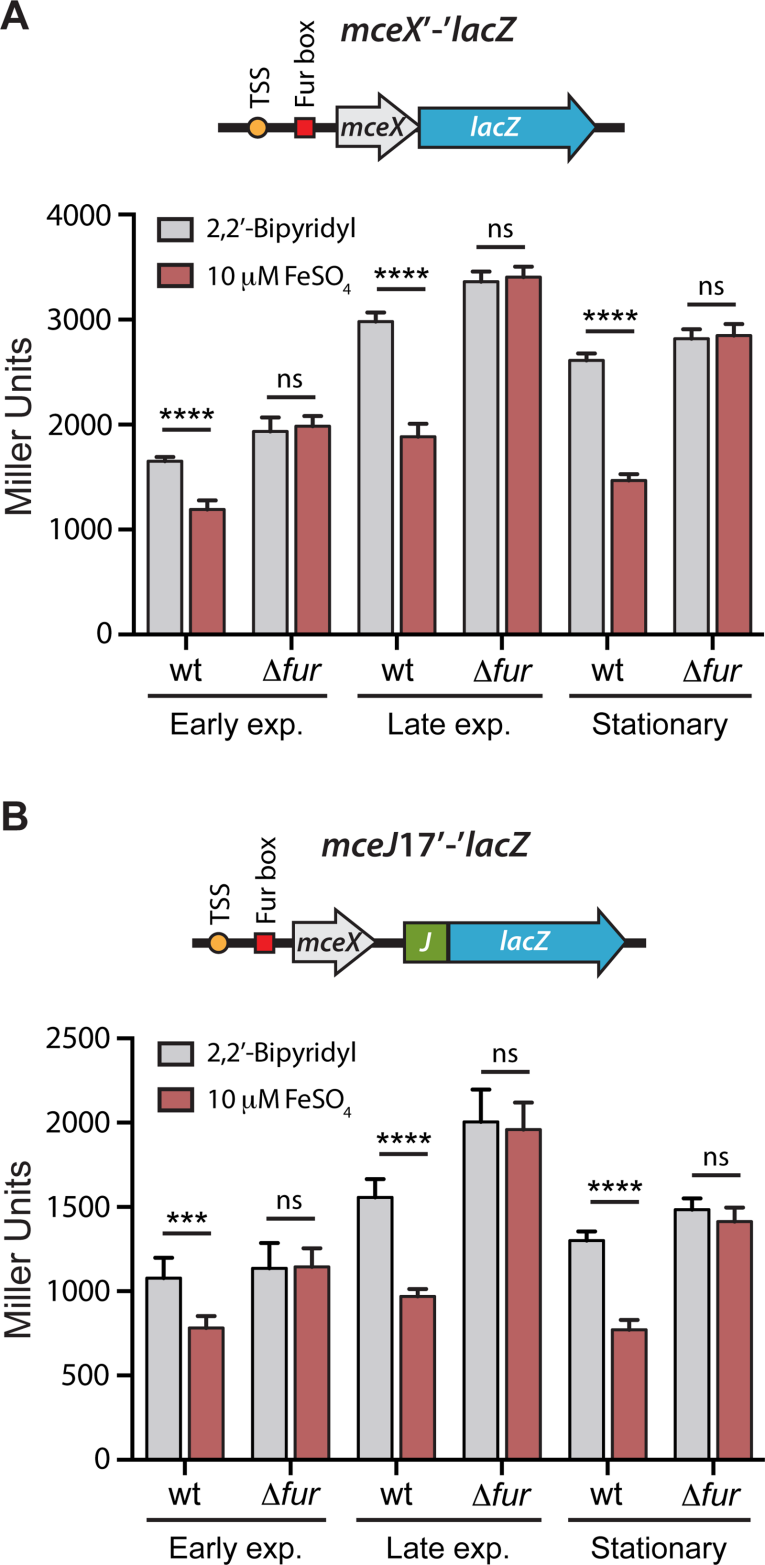
The expression of *mceX* and the microcin E492 maturation genes *mceJI* is regulated by Fur and iron availability. *wt* and Δ*fur E*. *coli* cells transformed with the reporters p*mceX*’-‘*lacZ* (A) or p*mceJ17*’-‘*lacZ* (B), were grown in M9 medium supplemented with 100 0μM 2,2’-Bipyridyl (low iron availability) or 10 μM FeSO_4_ (high iron availability). β-galactosidase activity (expressed in Miller Units) was measured at different phases of growth (Early exponential, OD_600_ = 0.4–0.6; Late exponential, OD_600_ = 0.9–1.1; Stationary, OD_600_ = 1.9–2.1). In the construct schemes, the orange circle represents the experimentally determined transcription start site and the red square represents the functional Fur box. Error bars represent the standard deviation between 6 independent experiments. ***P<0.001, ****P<0.0001, ns: not significant.

To investigate the role of iron availability and Fur in regulating *mceX* and *mceJI* expression, we built the reporter fusions *mceX*’-‘*lacZ* and *mceJ17*’-‘*lacZ*. The former comprises the promoter region of *mceX* (including the primary TSS and Fur box) and *lacZ* fused to the last codon of *mceX* coding region (Fig 3A), while the latter comprises the promoter and the coding region of *mceX*, and *lacZ* fused to the seventeenth *mceJ* codon (Fig 3B). Cells of *E*. *coli* MC4100 or its Δ*fur* derivative strain H1941, were transformed with the *mceX*’-‘*lacZ* or *mceJ17*’-‘*lacZ* fusions (cloned in p*mceX*’-‘*lacZ* and p*mceJ17*’-‘*lacZ*, respectively). LacZ activity was measured during bacterial growth under iron deprivation (100 μM 2,2’-Bipyridyl, BIP) or under high iron availability (10 μM FeSO_4_) (Fig 3). Both fusions showed a similar expression pattern in the wild type strain, with a higher LacZ activity in the presence of the iron chelator BIP. In presence of iron, such activity was reduced to ~70% in early exponential phase of growth, and to ~55–60% in late exponential and stationary phase. Conversely, the repressive effect of iron on the expression of both reporter fusions was not observed in the Fur-defective strain (Fig 3), providing additional evidence regarding the role of this regulator in the iron-mediated control of *mceX*-*mceJI* gene expression. In addition, overexpression of Fur led to a significant decrease in the reporter activity in both wt and Δ*fur* strains (not shown).

Previous reports demonstrated that the expression of *iroB*, the *mceC* homolog, is regulated by Fur [29,42]. Although a putative Fur box with a 58% identity with the *E*. *coli* consensus was found overlapping -10 element of the *mceC* promoter region, Fur-TA assays revealed that this element failed to bind Fur *in vivo* (Fig 1D). However, these assays are not quantitative and the possibility of a low affinity binding of Fur to the Fur box of *mceC* cannot be discarded. To corroborate that Fur does not act regulating *mceC* gene expression, we transformed *wt* and Δ*fur* strains with a construct harboring *lacZ* fused to the last codon of *mceC* gene (*mceC’-‘lacZ*), measuring the β-galactosidase activity over growth (S1 Fig). *mceC* expression remained unchanged at distinct growth stages in both *wt* and Δ*fur* strains, and the activity measured in the corresponding hosts showed no significant differences. Thus, we conclude that Fur does not regulate *mceC* expression, probably because it is unable to bind to the putative *mceC* Fur box *in vivo*.

### MceX is a regulator that acts repressing the expression of the MccE492 structural gene and its immunity protein

**Fig 4:**
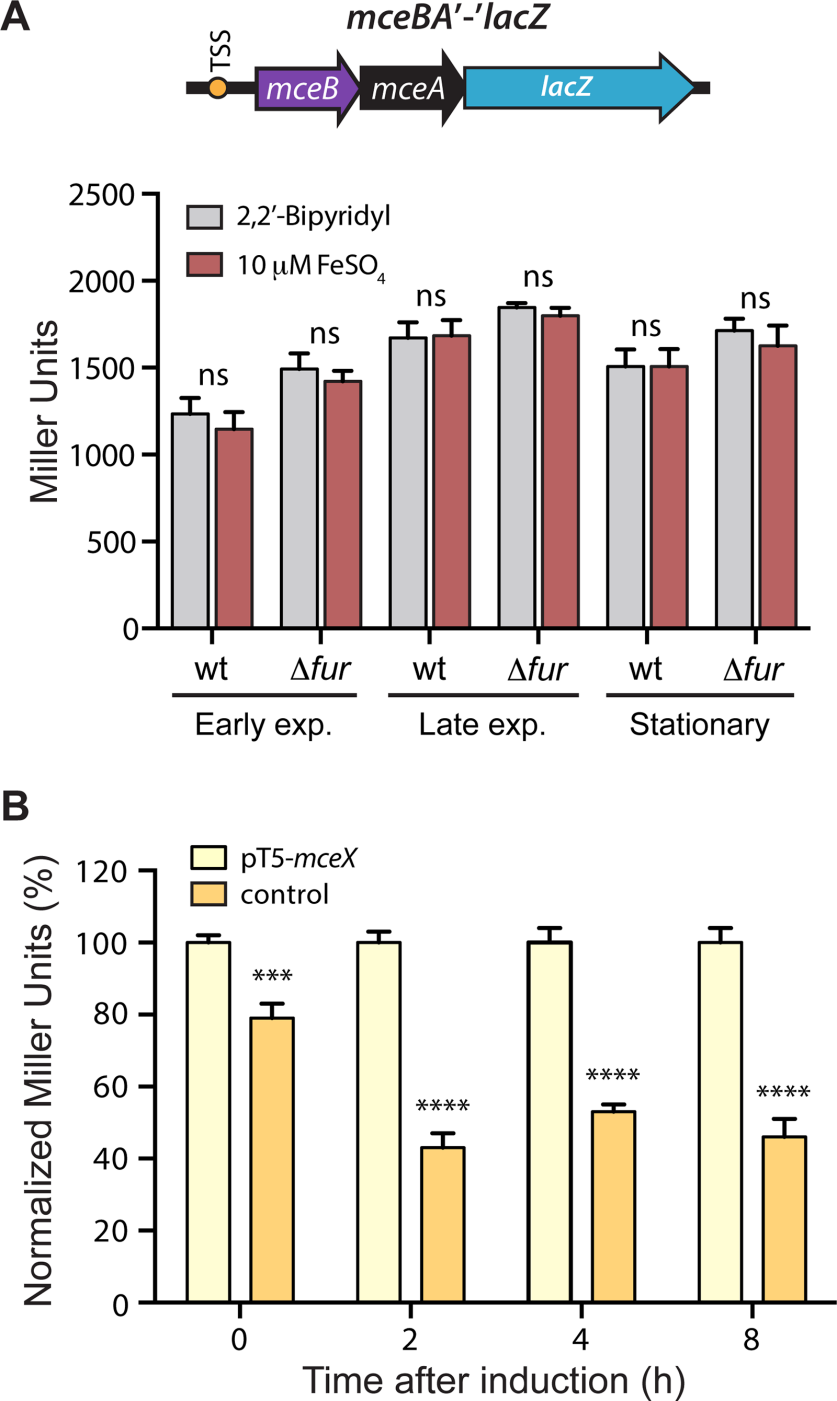
MceX negatively regulates the expression of the microcin E492 structural gene. (A) *wt* and Δ*fur E*. *coli* cells transformed with the reporter p*mceBA*’-‘*lacZ* were grown in M9 medium supplemented with 100 μM 2,2’-Bipyridyl (low iron availability) or 10 μM FeSO_4_ (high iron availability). β-galactosidase activity (expressed in Miller Units) was measured at different growth phases (Early exponential, OD_600_ = 0.4–0.6; Late exponential, OD_600_ = 0.9–1.1; Stationary, OD_600_ = 1.9–2.1). In the construct scheme, the orange circle represents the previously determined transcription start site. (B) Wild type *E*. *coli* cells were transformed with pT5-*mceX* (allowing the IPTG-inducible expression of MceX), or with the backbone plasmid pUC57 as negative control. The activity was expressed as the percentage of β-galactosidase activity after induction respect to the negative control. Error bars represent the standard deviation between 6 independent experiments. ***P<0.001, ****P<0.0001, ns: not significant.

The results presented above indicate that the previously uncharacterized gene *mceX* is translated, and that its expression is modulated by iron availability in a Fur-dependent mechanism. However, its role in the MccE492 production is unknown. According to an *in-silico* prediction, MceX would have a leucine zipper and one transmembrane domain, which is also present in its homolog MchX (68% similarity) from the microcin H47 (MccH47) system [43]. MchX function is also unknown, and its genetic organization in the MccH47 system is different than that of MccE492: while in MccH47 the *mchX* gene is encoded in the operon containing the structural and immunity genes *mchBI*, in MccE492 is located in the operon encoding the maturation genes *mceJI*. It was demonstrated that the expression of *mchX* is under Fur repression [6], and that it may act as a regulator of the expression of MccH47 structural gene [43]. Considering the similarities between MceX and MchX, we evaluated if MceX acts regulating the expression of the MccE492 structural gene *mceA*. We previously showed that *mceA* and *mceB* form a single transcriptional unit (*mceBA*) where 14 nucleotides of both coding regions overlap, and which promoter is located upstream of *mceB* [40]. In order to investigate the effect of iron and MceX over the expression of this transcriptional unit, we constructed the reporter fusion *mceBA*’-‘*lacZ*, consisting of the *mceBA* promoter region, and LacZ fused to the last amino acid residue of the MceA protein. Thus, the expression of this fusion depends mainly on the unit promoter and the ribosome binding site (RBS) of *mceA*. We first assessed if iron availability has a direct effect in the *mceBA* expression, measuring the LacZ activity of wild type or Δ*fur E*. *coli* cells carrying the *mceBA*’-‘*lacZ* fusion, under iron deprivation or under high iron availability (Fig 4A). Consistent with the lack of a functional Fur box in its promoter region (Fig 1), the expression of *mceBA* showed to be independent of the iron availability and of the presence of the Fur regulator.

We next evaluated the direct effect of MceX in the expression of the fusion. For this purpose, we transformed wild type *E*. *coli* cells carrying p*mceBA*’-‘*lacZ* with pT5-*mceX*, a compatible plasmid that allows the IPTG-inducible expression of *mceX*. LacZ activity was measured at 2, 4, and 8 h after induction, and compared with that registered from *E*. *coli* cells carrying p*mceBA*’-‘*lacZ* plus a control plasmid (Fig 4B). MceX overexpression led to a significant decrease in the LacZ levels in all the times evaluated, indicating that this protein effectively acts as a negative regulator of the *mceBA* expression.

### Fur and MceX modulate the antibacterial activity of *E*. *coli* cells carrying MccE492 production systems

**Fig 5:**
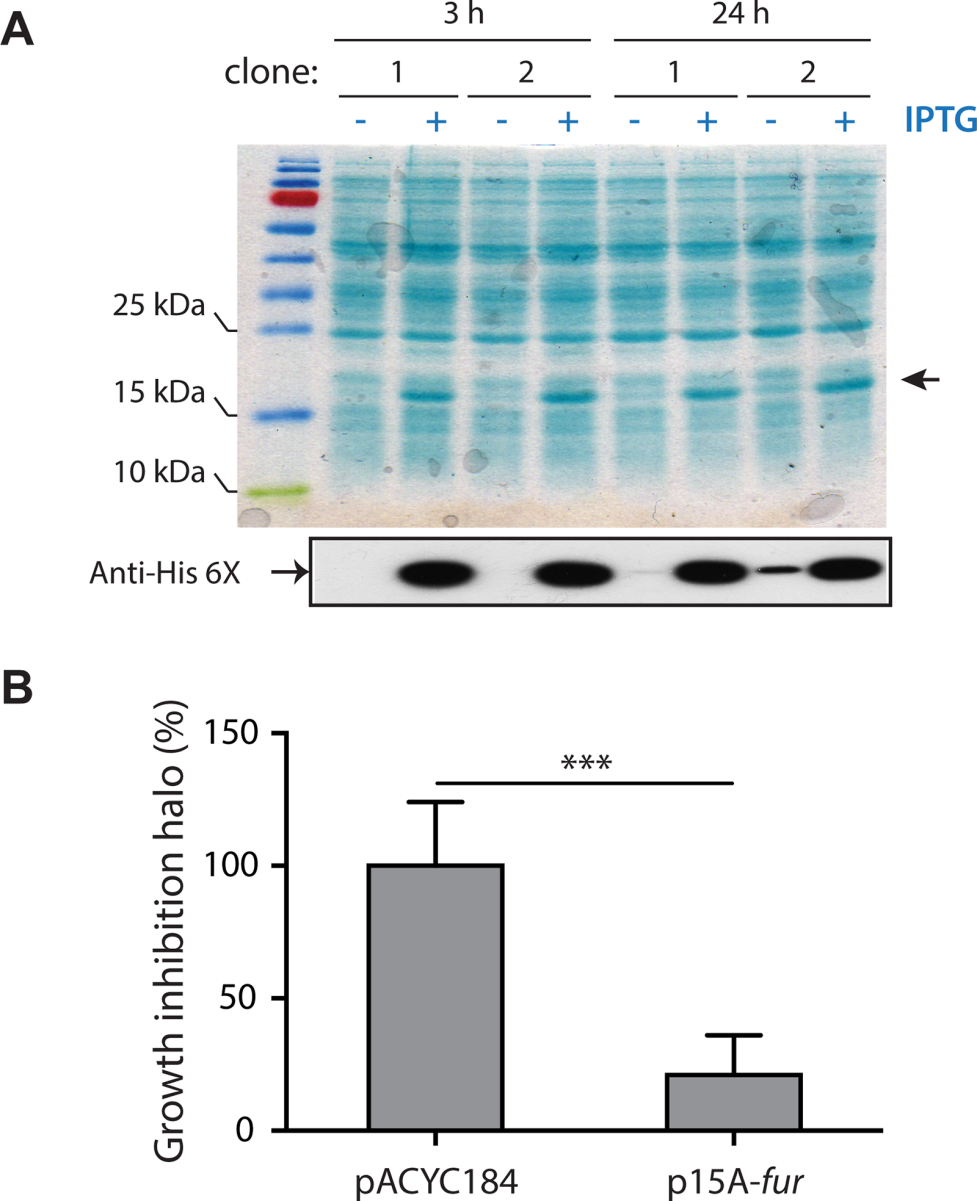
Fur overexpression reduces the antibacterial activity of *E*. *coli* cells carrying the gene cluster for microcin E492 production. *E*. *coli* cells carrying pMccE492 (allowing the production of active MccE492) were transformed with p15A-*fur* (driving overexpression of Fur carrying a His-tag) or pACYC184 (control). (A) Two representative clones of p15A-*fur* containing cells were grown in M9 medium alone or supplemented with IPTG (1 mM), and total protein extracts were prepared after 3 and 24 h of growth. SDS-PAGE analysis of the extracts followed by Coomassie Blue staining revealed a ~17-kDa protein band largely enriched in cells grown in presence of IPTG (black arrow). Immunoblot using an anti-His antibody confirmed the induction of Fur expression in presence of IPTG, although a basal expression was detected after 24 h of growth in absence of inducer. (B) Antibacterial activity of *E*. *coli* cells producing MccE492, transformed with p15A-*fur* or a control plasmid (pACYC184). The antibacterial activity was measured as a function of the growth inhibition halo’s area over a sensitive strain layer, in M9 medium supplemented with IPTG, and then normalized to the value obtained in the control condition (plasmid backbone). Error bars indicate standard deviation from 40 measurements performed for each condition. ***P<0.001.

The results described above demonstrated that the expression of *mceX* and *mceJI* genes is repressed by increasing iron concentrations in a Fur-dependent mechanism. MccE492 toxic activity requires posttranslational attachment of a salmochelin-like moiety in a reaction catalyzed by the MceJI gene products. Consequently, Fur overexpression should lead to a decrease in the antibacterial activity of *E*. *coli* cells producing MccE492. To test this hypothesis, we constructed the p15A-*fur* plasmid, a pACYC184-derivative harboring a 6XHis-tagged *fur* gene under the control of T5 promoter. This plasmid allows the IPTG-inducible expression of Fur and is compatible with pMccE492. Wild type *E*. *coli* cells carrying pMccE492 were transformed with either p15A-*fur* or pACYC184 (control). After transformation, we corroborated Fur overexpression in cells carrying p15A-*fur* plasmid, by growing them in liquid medium until exponential phase and inducing the expression with IPTG. Total protein extracts were prepared after 3 h and 24 h of induction and analyzed by SDS-PAGE (Fig 5A). A ~17-kDa protein band, compatible with the previously determined Fur molecular weight (16.8 kDa) [44–45], was enriched in the IPTG-treated samples. Immunoblot analysis using an anti 6XHis-tag antibody confirmed that the enriched band corresponds to Fur protein expressed from p15A-*fur*. Although in a much lesser extent, this protein was also detected in the non-induced samples, indicating some leaky expression from the T5 promoter (more evident at 24 h post-induction).

To test the effect of Fur overexpression on MccE492 activity, several colonies of each strain (carrying pMccE492 plus p15A-*fur* or the control plasmid) were stabbed into a top-agar lawn of MccE492-sensitive *E*. *coli* cells, in presence of IPTG. After overnight incubation at 37 °C, the antibacterial activity was measured as a function of the area of the growth-inhibition halos generated by each colony (Fig 5B). The antibacterial activity was significantly reduced in cells overexpressing Fur, compared with cells carrying the control plasmid. These results indicate that Fur inhibits mature MccE492 production, and this can be explained at least in part by the down-regulation of the *mceJI* expression.

**Fig 6:**
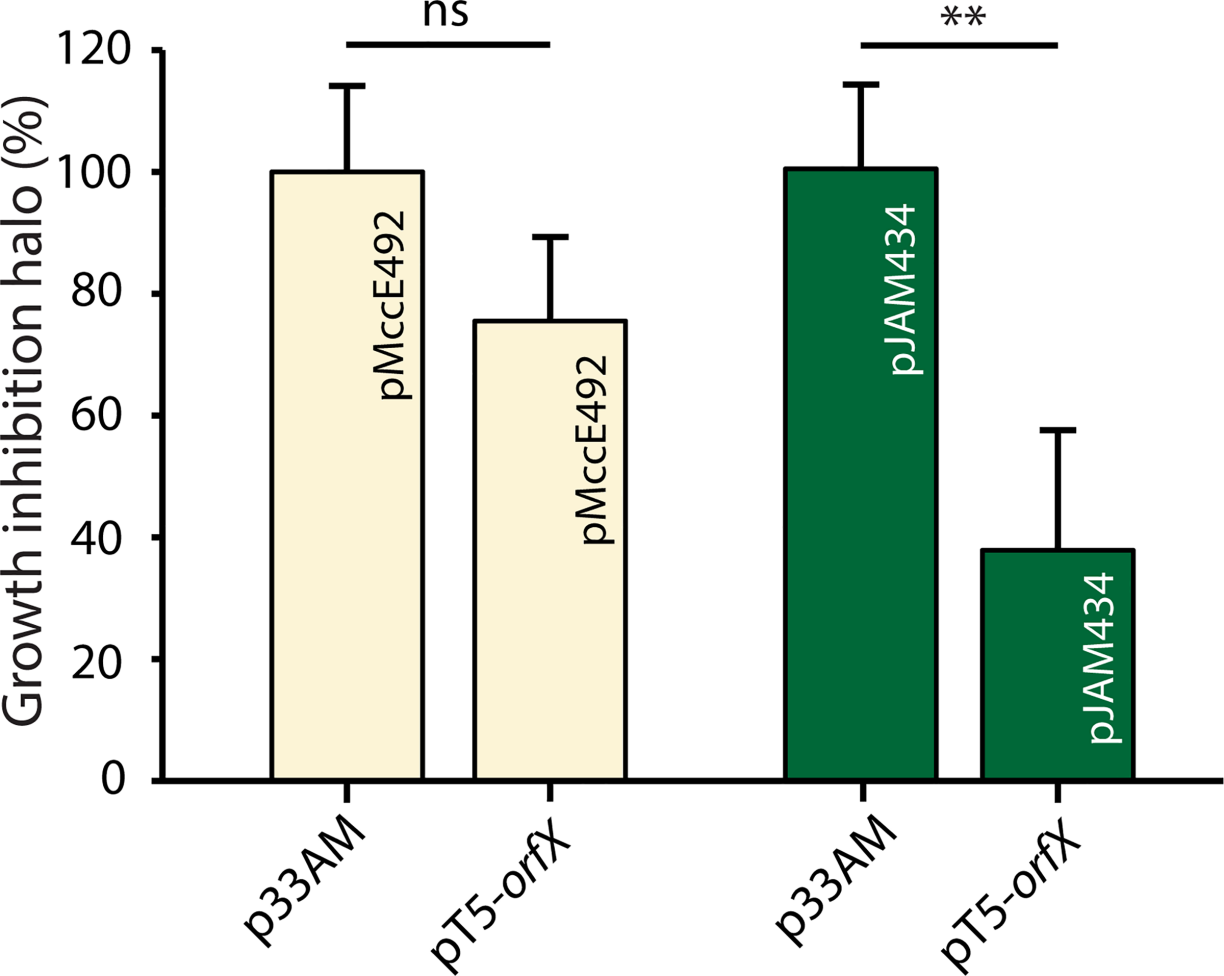
MceX overexpression reduces the antibacterial activity of *E*. *coli* cells carrying the gene cluster for microcin E492 production. *E*. *coli* cells carrying pMccE492 (wt, in yellow) or pJAM434 (poor producing, in green) were transformed with pT5-*mceX* (driving overexpression of MceX) or pUC57 (control). Antibacterial activity was measured as a function of the growth inhibition halo’s area over a sensitive strain layer, in M9 medium supplemented with IPTG, and was normalized to the value obtained in the control condition for each case. Error bars indicate standard deviation from 40 measurements performed for each condition. **P<0.01, ns: not significant.

A second regulatory circuit connecting MccE492 production and iron availability would result from the action of MceX on the expression of the *mceBA* unit. Thus, we tested the effect of MceX overexpression on the production of active MccE492. With this purpose, we transformed *E*. *coli* cells carrying the MccE492 production systems pMccE492 or pJAM434, with the compatible plasmid pT5-*mceX*. pMccE492 harbors all the genes required for the production of active MccE492, as present in the *K*. *pneumoniae* RYC492 chromosome, producing a high activity of this microcin. On the other hand, pJAM434 also allows the production of active MccE492, but due to an inversion of a DNA segment containing *mceX* and the maturation genes, produces a truncated version of MceX and a low expression of the other genes that results in a poor producing strain [17]. The cells transformed with each couple of plasmids (pT5-*mceX* plus pMccE492 or pJAM434) were spotted into agar plates supplemented with IPTG, containing a lawn of a MccE492-sensitive strain. The antibacterial activity was measured as a function of the area of the growth-inhibition halos generated by each colony, which was normalized by the activity registered for the control plasmid in each case (Fig 6). Overexpression of MceX in cells carrying pMccE492 caused a slight decrease in the antibacterial activity (not significant), suggesting that the MceX protein produced from the wild type pMccE492 is enough for the repressive effect, and thus mask the effect of the protein expressed from the inducible plasmid pT5-*mceX*. Conversely, when MceX is overexpressed in cells carrying the pJAM434 system (harboring a disrupted copy of *mceX*), we observed a significant decrease in the antibacterial activity, compared to the cells transformed with the control plasmid. These results are in agreement with the role of MceX in repressing the expression of the MccE492 structural gene.

## Discussion

The evidence provided in this study indicates that iron metabolism and MccE492 maturation are processes coordinately regulated. The expression of the two essential components for MccE492 posttranslational modification MceJ and MceI, and thus the amount of active MccE492 produced, is modulated in response to iron availability through the action of the ferric uptake regulator Fur. Moreover, our results indicate that the iron supply also exerts an indirect control of the MccE492 structural gene expression, through the action of the negative regulator MceX.

Despite this regulatory circuit was studied in an *E*. *coli* host and the MccE492 production cluster was originally isolated from a strain of *Klebsiella pneumoniae*, different evidence support that the conclusions obtained herein would also be valid for *Klebsiella*. First, several examples Fur-regulated genes, including some related with type 3 fimbriae assembly and capsular polysaccharide biosynthesis, have been reported in different isolates of *Klebsiella pneumoniae* [46–50]. In addition, the search for a *fur* gene in the *K*. *pneumoniae* RYC492 genome [41], revealed that a full-length *fur* coding region is present. This ORF encodes for a 150 amino acids Fur protein with 96% identity to *E*. *coli* Fur (148 amino acids). The high level of identity is compatible with the binding of both *E*. *coli* and *K*. *pneumoniae* Fur to the same DNA elements.

The search for Fur boxes along the MccE492 genetic cluster revealed the presence of several putative DNA elements with more than 50% identity with the *E*. *coli* Fur box consensus, located in the promoter region of *mceC*, *mceD*, *mceE* and *mceX*/*mceJI* genes. However, FurTA assays showed that only the Fur boxes from the intergenic region between *mceE* and *mceX* bind Fur *in vivo*. This correlates with the fact that *mceE* and *mceX* boxes showed the highest identity with the *E*. *coli* consensus (68% and 74%, respectively), while those from *mceC* and *mceD* genes showed 58% and 63% identity, respectively. Furthermore, *mceX* Fur box harbors all the eight most conserved bases determined for this binding site, proposed to be the main determinants for Fur regulator binding in *E*. *coli* [25, 51].

The RT-PCR studies demonstrated that the transcription of *mceJI* genes is coupled to *mceX*, and thus is subject to Fur regulation through *mceX* Fur box. This is the first study confirming the expression of this ORF, which has homologs in other chromosome-encoded microcin systems [6,43]. Using *LacZ* fusions, we corroborated that this ORF is translated, and that its overexpression led to a significant decrease in the expression of the *mceBA* genes. In order to further support that the decreased LacZ activity observed after MceX overproduction is not due to the titration of the transcriptional/translational machineries upon overexpression of any gene, we tried the effect of MceX overexpression on its own synthesis, and we did not find any effect (S2 Fig). Moreover, we showed that MceX overexpression reduced the antimicrobial activity of MccE492-producing *E*. *coli* cells. The role of MceX is consistent with the function of MchX (the MceX homolog in the microcin H47 system), which was proposed to regulate the MccH47 structural gene. Here, *mchX* has a functional Fur box in its promoter region, which mediates Fur-dependent repression of this gene at high iron concentrations [6]. This was proposed as an explanation of why active MccH47 production is low when iron is abundant. However, in this system *mchX* seems to be co-transcribed with the immediately downstream genes *mchI* and *mchB*, coding for the microcin H47 immunity and the bacteriocin protein, respectively [43]. Posttranslational modification genes *mchC* and *mchD* (82% and 95% similarity with *mceJ* and *mceI*, respectively) lay 270 bp downstream *mchB*. The disruption of *mchX* gene by transposon insertions caused a decrease in the MccH47 activity. Although the mechanism underlying such effect was not directly addressed, based on the analysis of several mutants it was proposed that i) *mchX* is co-transcribed with at least one gene that participates in MccH47 production, so transposon insertion in *mchX* disrupts the transcriptional flux through downstream genes, and or ii) MchX has a regulatory role, acting as an activator of some of the MccH47 production genes and inducing its own expression [43]. Consequently, iron-dependent Fur-mediated transcriptional repression of *mchX* should cause a reduction in the MccH47 activity by downregulating at least the *mchB* gene. Since the expression of catechol receptors is highly induced when iron levels are low, Patzer and coworkers [6] proposed that induction of MccH47 production under low iron conditions would be a mechanism to select against bacteria competing for the same siderophore. However, for this mechanism to be effective, the production of active MccH47 at low iron concentrations should require also the up-regulation of maturation genes, as demonstrated to occur in the MccE492 system. Accordingly, a regulatory effect of iron levels and Fur over the expression of MccH47 maturation genes cannot be discarded, because it is possible that the polycistronic transcript harboring *mchXIB* extends beyond to include *mchC* and *mchD* genes, putting them under the control of *mchX* Fur box.

Regarding *mceC* maturation gene, although a putative Fur box was identified in its promoter region, FurTA assay discarded *in vivo* binding of this regulator. Consistently, a constitutive-expression profile was observed using the *mceC’-‘lacZ* fusion, and no differences were seen comparing the expression levels in a *wt* and a Δ*fur* background. Conversely, iron levels (through the action of Fur) regulate the expression of *mceC* homolog *iroB* from *Salmonella enterica* [29]. This points out the different evolutionary history of glycosyltransferase genes from *iroBCDEN* and those harbored by chromosomal microcin-production clusters, where this function is not regulated by Fur, as demonstrated for MccE492 (this study) and MccH47 [6]. Constitutive *mceC* expression indicates that the production of salmochelin-like molecules starting from enterochelin is available in all stages of growth. This probably means a competitive advantage for cells carrying the MccE492 production system, since salmochelin evades the iron-sequestering protein lipocalin present in mammal’s serum, which binds and neutralize enterochelin [20]. In this line, previous evidences indicate that salmochelin and or MccE492 likely play a role in *K*. *pneumoniae* pathogenesis. First, determinants for the production of these molecules were highly associated with hypervirulent strains [9–10]. Second, the MccE492 production cluster forms part of an unstable and possibly mobilizable genomic island (harboring a putative conjugal transfer origin), which was highly associated with strains isolated from liver abscesses [11]. Consistent with this, in a recent work we showed that *K*. *pneumoniae* RYC492 (harboring the Mcc492 production cluster in its chromosome) is highly virulent for zebrafish larvae and *Dictyostelium discoideum* infection models [52]. Additionally, K1 hypervirulent isolates were shown to carry a higher frequency of genes associated with salmochelin production, among an ethnically diverse population evaluated as part of the Antibiotics for *Klebsiella* Liver Abscess Syndrome Study (A-KLASS) clinical trial in Singapore [53]. Moreover, salmochelin and yersiniabactin siderophore production and its interaction with Lipocalin 2 determined the replicative niche of *Klebsiella pneumoniae* in a mice Pneumonia model [54]. However, other evidence studying the siderophore repertoire of this species indicated that aerobactin, but not yersiniabactin, salmochelin, or enterobactin, is determinant for the growth and survival of hypervirulent *K*. *pneumoniae ex vivo* and *in vivo* [55]. Thus, additional studies must be performed to understand the contribution of both MccE492 and salmochelin production for the pathogenesis of this species.

On the other hand, the FurTA assays revealed that there is a functional Fur box in the promoter region of *mceE*. The meaning of a putative Fur-mediated regulation of *mceE* gene is unclear, since the function of MceE protein remains uncharacterized. However, some clues arise from the analysis of its amino acid sequence. The C-terminal portion of MceE shares some degree of identity with MchS4, a protein encoded in the MccH47 system, which promotes enterochelin production through an unknown mechanism [56]. Hence, it is possible that some components of microcin production systems like MceE ensure an adequate supply of enterochelin, to participate as a substrate for microcin maturation.

To address the effect of Fur over the active MccE492 production, we first attempted to compare active MccE492 production in both *wt* and Δ*fur* backgrounds. However, several attempts failed to obtain Δ*fur* cells transformed with pMccE492 or pJAM229 systems, and we could only accomplish the transformation with pJAM434, the low producing variant. This suggests that *mceJI* overexpression in absence of Fur can be deleterious or toxic in MccE492 producing cells. For this reason, we tried the opposite, i.e. to evaluate the effect of *fur* overexpression over the active MccE492 production. These results showed that *fur* overexpression led to a decrease in the antibacterial activity of cells producing MccE492. These results are in agreement with the findings of Vassiliadis *et al*. [57] who reported the absence of mature MccE492 production at high iron levels. This effect can now be explained by the contribution of at least two regulatory pathways. First, as demonstrated in this work, in the presence of iron Fur acts directly repressing the expression of maturation genes *mceJI*, so less mature microcin would be produced. Additionally, at high iron levels Fur inhibits enterochelin biosynthesis, binding to Fur boxes located in the promoter of several genes of that pathway and causing their repression [26,58]. Other more distant examples of Fur and iron-mediated regulation of microcin production are colicin V [59] and MccJ25 [60]. Deprivation of iron elicited MccJ25 production, but studies in a fur background still presented some repression by iron [60]. In the case of colicin V, the transcription of two diverging operons comprising the genes necessary for its production is repressed by Fur under high iron levels conditions (reviewed in [61]). The expression of those genes is induced at the end of the exponential phase of growth as a result of the concomitant iron depletion [62]. Interestingly, colicin V also requires the siderophore receptor Cir to cross the outer membrane to exert its toxic effect, supporting the hypothesis that induction of active microcin production at low iron levels relates to killing bacteria competing for the same siderophores, as proposed by Patzer and coworkers [6].

**Fig 7:**
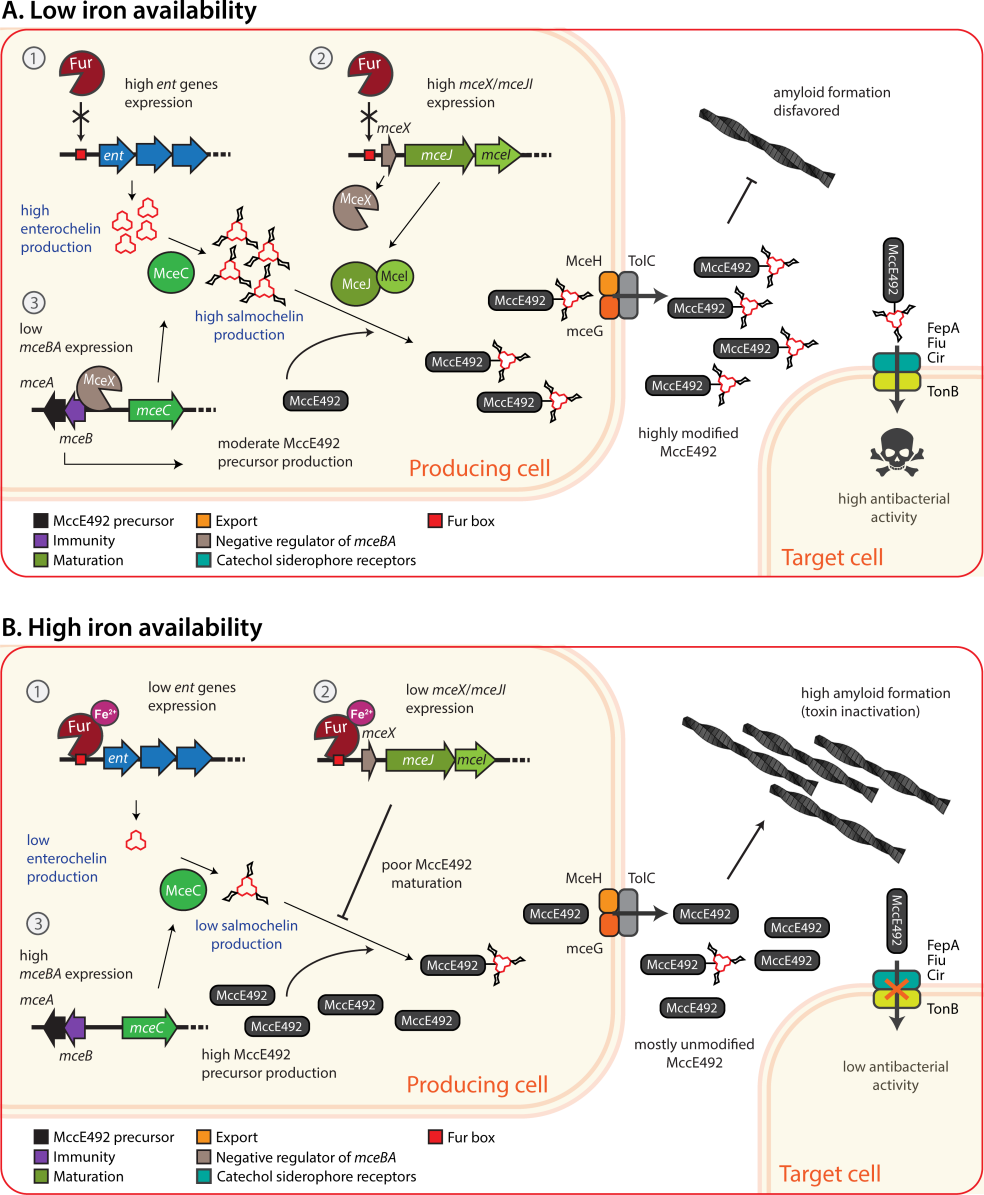
Model of the iron regulatory circuit controlling microcin E492 production, maturation and antibacterial activity. (A) At low iron availability, no Fur-Fe^2+^ repressor complexes are available to bind the Fur box located in the promoters of genes participating in the synthesis of enterochelin (1) and *mceX*/*mceJI* genes (2). Thus, high amounts of enterochelin and salmochelin are produced, and MceJI proteins catalyze the attachment of salmochelin to MccE492 peptide (maturation). Additionally, the MceX regulator partially represses the *mceBA* genes (3), restricting the production of immature MccE492. In this situation, a high proportion of modified MccE492 is exported, which can enter the target cells through the catechol siderophore receptors, resulting in a high antibacterial activity. The production of a high amount of modified MccE492, likely disfavors its amyloid aggregation, preventing toxin inactivation. (B) At high iron availability, the Fur-Fe^2+^ complexes repress the *ent* and *mceX*/*mceJI* genes, causing a low production of enterochelin and salmochelin, a poor MccE492 maturation process, and the release of the negative regulation exerted by MceX over the *mceBA* genes, allowing a higher expression of the MccE492 precursor. Hence, unmodified MccE492 is predominantly exported, which is not recognized by the siderophore receptors and thus fails to cause a toxic effect. The high proportion of unmodified MccE492 likely favor its aggregation into amyloid fibers, and thus the loss of antibacterial activity. The scheme includes a simplified representation of the actual *ent* genes organization.

Overall, the evidence provided in this work supports a model for the iron regulatory circuit controlling MccE492 production, maturation and likely amyloid formation, as described in Fig 7. When iron is scarce (Fig 7A), no Fur-Fe^2+^ complexes are available to act as repressors. Consequently, Fur fails to bind the Fur boxes located in the promoters of several genes participating in the synthesis of enterochelin (*ent* genes; “1” in the schemes of Fig 7A and 7B), allowing their expression and thus the production of high amounts of enterochelin. Subsequently, the constitutively expressed MceC protein catalyzes the glycosylation of enterochelin producing salmochelin. Besides, Fur is unable to repress the *mceX*/*mceJI* genes (“2” in the schemes of Fig 7A and 7B) and thus MceJ and MceI are available to catalyze the attachment of salmochelin to MccE492 molecules. The export genes mceHG are part of this operon and consequently transcribed from the same promoter, but also from and independent promoter located upstream of *mceH* [15], so MccE492 export is not a limiting step. Additionally, MceX regulator lowers the expression of *mceBA* genes (“3” in the schemes of Fig 7A and 7B), restricting the production of immature MccE492. In this scenario, a high proportion of modified (active) MccE492 is exported, which can then enter to the periplasm of target cells through the catechol siderophore receptors and exert their toxic effect. In contrast, at iron plenty (Fig 7B), the Fur-Fe^2+^ complexes bind to the fur boxes of the *ent* and *mceX*/*mceJI* genes, lowering their expression. Thus, there is a low production of enterochelin and salmochelin, and a poor MccE492 maturation process. Also, low expression of the MceX negative regulator allows a higher expression of the MccE492 precursor. Hence, unmodified MccE492 is predominantly exported, which is not recognized by the siderophore receptors and thus fails to cause a toxic effect. As previously shown, modified forms of MccE492 have a decreased aggregation propensity compared to the unmodified peptide, which is highly amyloidogenic *in vitro* [5]. Thus, it is possible that iron abundance imposes a double negative control of MccE492 antibacterial activity. First, disfavoring the maturation process, and second increasing the production of MccE492 precursor and thus the unmodified/modified forms ratio, which in turn increase the amyloid formation propensity. This could be a way of promoting a rapid toxin inactivation in response to increasing iron abundance.

## Supporting information

S1 Fig Fur does not regulate *mceC* expression. *E*. *coli wt* and Δ*fur* cells were transformed with a reporter fusion between the last codon of *mceC* and *lacZ* (*mceC*’-‘*lacZ*), and growth in M9 medium. The optical density at 600 nm (A) and the β-galactosidase activity (B) were measured for each condition at different times and phases of growth (Early exponential, OD_600_ = 0.4–0.6; Late exponential, OD_600_ = 0.9–1.1; Stationary, OD_600_ = 1.9–2.1). Error bars correspond to standard deviation of three independent experiments.
(PDF)

S2 Fig Effect of MceX overexpression on the LacZ activity of cells carrying a *mceX’-‘lacZ* fusion. *E*. *coli* cells carrying a plasmid harboring lacZ gene fused to the first codon of *mceX* were transformed with the compatible plasmid pT5-*mceX*, allowing the IPTG-inducible expression of *mceX*. LacZ activity was measured for cells growing in presence of 1 mM IPTG or without IPTG, at different growth phases. *mceX* overexpression had no effect on the LacZ activity of the *mceX*’-‘*lacZ* fusion. Error bars correspond to standard deviation of three independent experiments.
(PDF)
