# Supplementary material for: The Ferric uptake regulator (Fur) and iron availability control the production and maturation of the antibacterial peptide microcin E492
Source: PLoS One. 2018 Aug 2;13(8):e0200835. doi: 10.1371/journal.pone.0200835 (PMC6071977; doi:10.1371/journal.pone.0200835)
Supplement: S1 Fig — E. coli wt and Δfur cells were transformed with a reporter fusion between the last codon of mceC and lacZ (mceC’-‘lacZ), and growth in M9 medium. The optical density at 600 nm (A) and the β-galactosidase activity (B) were measured for each condition at different times and phases of growth (Early exponential, OD600 = 0.4–0.6; Late exponential, OD600 = 0.9–1.1; Stationary, OD600 = 1.9–2.1). Error bars correspond to standard deviation of three independent experiments. (PDF) [file pone.0200835.s001.pdf]

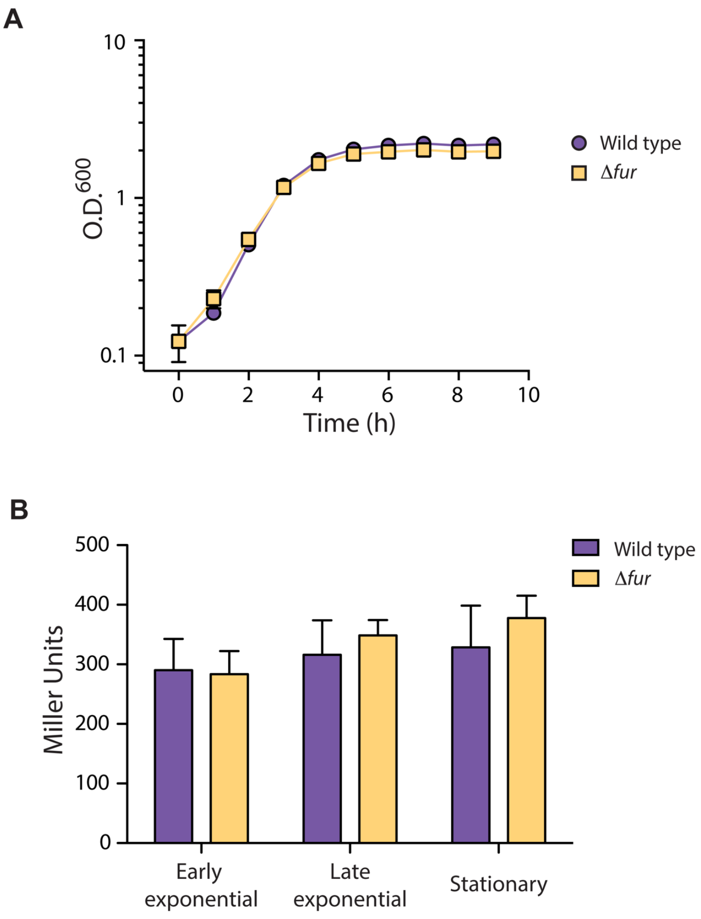

S1 Fig. Fur does not regulate *mceC* expression. *E. coli wt* and Δ*fur* cells were transformed with a reporter fusion between the last codon of *mceC* and *lacZ* (*mceC*'-'*lacZ*), and growth in M9 medium. The optical density at 600 nm (A) and the β-galactosidase activity (B) were measured for each condition at different times and phases of growth (Early exponential, OD_{600}=0.4-0.6; Late exponential, OD_{600}=0.9-1.1; Stationary, OD_{600}=1.9-2.1). Error bars correspond to standard deviation of three independent experiments.
