# Supplementary material for: The Ferric uptake regulator (Fur) and iron availability control the production and maturation of the antibacterial peptide microcin E492
Source: PLoS One. 2018 Aug 2;13(8):e0200835. doi: 10.1371/journal.pone.0200835 (PMC6071977; doi:10.1371/journal.pone.0200835)
Supplement: S2 Fig — E. coli cells carrying a plasmid harboring lacZ gene fused to the first codon of mceX were transformed with the compatible plasmid pT5-mceX, allowing the IPTG-inducible expression of mceX. LacZ activity was measured for cells growing in presence of 1 mM IPTG or without IPTG, at different growth phases. mceX overexpression had no effect on the LacZ activity of the mceX’-‘lacZ fusion. Error bars correspond to standard deviation of three independent experiments. (PDF) [file pone.0200835.s002.pdf]

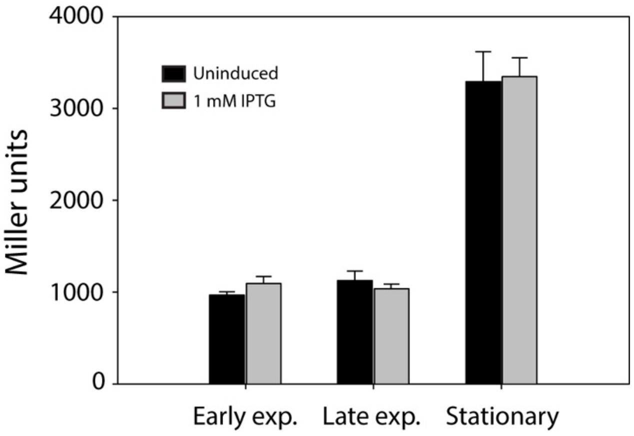

S2 Fig. Effect of MceX overexpression on the LacZ activity of cells carrying a *mceX'-'lacZ* fusion. *E. coli* cells carrying a plasmid harboring lacZ gene fused to the first codon of *mceX* were transformed with the compatible plasmid pT5-*mceX*, allowing the IPTG-inducible expression of *mceX*. LacZ activity was measured for cells growing in presence of 1 mM IPTG or without IPTG, at different growth phases. *mceX* overexpression had no effect on the LacZ activity of the *mceX*'-'*lacZ* fusion. Error bars correspond to standard deviation of three independent experiments.
